# Engineering xylose metabolism for production of polyhydroxybutyrate in the non-model bacterium *Burkholderia sacchari*

**DOI:** 10.1186/s12934-018-0924-9

**Published:** 2018-05-15

**Authors:** Linda P. Guamán, Carlos Barba-Ostria, Fuzhong Zhang, Edmar R. Oliveira-Filho, José Gregório C. Gomez, Luiziana F. Silva

**Affiliations:** 1 Universidad Tecnológica Equinoccial, Centro de Investigación Biomédica, Facultad de Ciencias de la Salud Eugenio Espejo, Quito, Ecuador; 2 Department of Microbiology, Institute of Biomedical Sciences, University of São Paulo, Av Prof Lineu Prestes 1374 Lab 148, Cidade Universitária, São Paulo, SP 05508-888 Brazil; 3 Faculty of Science and Food Engineering, Technical University of Ambato, Ambato, Ecuador; 4 Faculty of Health Sciences, Technical University of Ambato, Ambato, Ecuador; 5 Department of Energy, Environmental and Chemical Engineering, Washington University in St. Louis, Saint Louis, MO 63130 USA

**Keywords:** *Burkholderia*, Xylose, P(3HB), *xylR*, BglBricks

## Abstract

**Background:**

Despite its ability to grow and produce high-value molecules using renewable carbon sources, two main factors must be improved to use *Burkholderia sacchari* as a chassis for bioproduction at an industrial scale: first, the lack of molecular tools to engineer this organism and second, the inherently slow growth rate and poly-3-hydroxybutyrate [P(3HB)] production using xylose. In this work, we have addressed both factors.

**Results:**

First, we adapted a set of BglBrick plasmids and showed tunable expression in *B. sacchari*. Finally, we assessed growth rate and P(3HB) production through overexpression of xylose transporters, catabolic or regulatory genes. Overexpression of *xylR* significantly improved growth rate (55.5% improvement), polymer yield (77.27% improvement), and resulted in 71% of cell dry weight as P(3HB).

**Conclusions:**

These values are unprecedented for P(3HB) accumulation using xylose as a sole carbon source and highlight the importance of precise expression control for improving utilization of hemicellulosic sugars in *B. sacchari*.
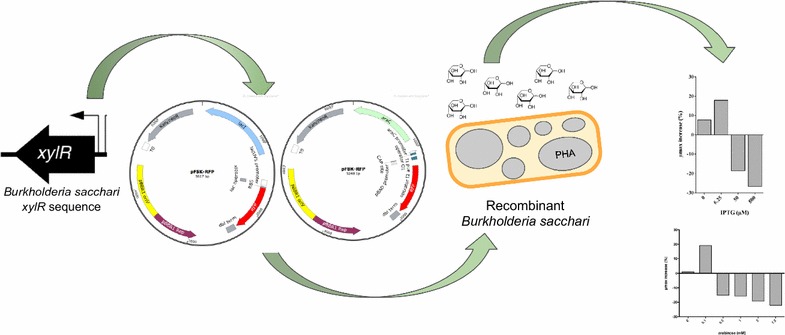

## Background

Lignocellulose consisting of cellulose, hemicellulose, and lignin, is the most abundant renewable resource worldwide [1]. Utilization of xylose, the second most abundant sugar in hemicellulosic materials, is essential for the efficient conversion of biomass to high-value chemicals. Some bacteria are known to efficiently transform xylose to polyhydroxyalkanoates (PHA), however, the rates and yields need to be improved to make this biopolymer production economically viable. One potential option to accomplish this is to integrate PHA production to a sugarcane mill [2, 3]. PHAs are accumulated as intracellular granules by several bacterial strains when a carbon source is provided in excess with respect to other essential nutrients (e.g., N, P, K, Mg, Fe, among others) [4]. These renewable biopolymers display properties (biodegradability, biocompatibility, low cytotoxicity) that make them ideal substitutes for oil derived plastics [5–11]. Strategies to improve microbial production using xylose often include bioprocess optimization [12], overexpression of xylose transporters [13, 14] or catabolic proteins [15, 16]. In recent years, the application of synthetic biology approaches in microbial platforms has opened new possibilities for engineering, optimization [17, 18] or even the re-design of metabolic pathways in *Escherichia coli, Saccharomyces cerevisiae,* and other model organisms. BglBrick vectors, one of the most widely used expression platforms in synthetic biology, have been used successfully to engineer native and synthetic pathways allowing the production of biofuels, bioplastics precursors, pharmaceuticals and other high-value chemicals [19, 20]. Despite these significant advances, the potential for production at industrial scale is still limited by the use of expensive raw materials, precursors, or the conversion of non-renewable feedstocks, among other factors.

*Burkholderia sacchari* LMG 19450 LFM101 is a Gram-negative bacterium, isolated from sugarcane crops in Brazil [21, 22], with potential for industrial-scale production of high-value molecules (e.g., xylonic acid, xylitol, and poly-3-hydroxybutyrate [P(3HB)]) from glucose, sucrose, xylose, arabinose, and other renewable carbon sources [12, 23]. Additionally, this bacterium can accumulate up to 80% cell dry weight as P(3HB) from sucrose [3] and produce hybrid PHA copolymers (incorporating hydroxyalkanoate monomers other than 3-hydroxybutyrate) [24–26]. Despite this great potential, the lack of molecular tools available for this organism and the inherently slow growth rate on xylose (0.15 h^−1^) must be improved to allow its use for industrial-scale production [27]. In the present study, two BglBrick plasmids were successfully adapted to control protein expression in the non-model bacterium *B. sacchari.* The constructed plasmids were used to individually overexpress all transporters (*xylE* and *xylFGH*), catabolic (*xylA* and *xylB*) and regulatory (*xylR*) genes, involved in xylose utilization through the xylose isomerase pathway, and to evaluate its individual effects on growth rate and production of P(3HB). Expression of appropriate levels of the xylose transcriptional regulator (*xylR*) enhanced both growth rate and P(3HB) production using xylose as the only carbon source. To our knowledge, this paper represents the first successful attempt to improve growth rate and P(3HB) through overexpression of catabolic and regulator genes in *B. sacchari.* This work emphasizes the value of developing genetic tools which allow precise and tunable control of expression in non-model organisms.

## Materials and methods

### Experimental procedures

#### Chemicals and media

Unless otherwise specified, all chemicals were obtained from Sigma-Aldrich^®^ (Sigma-Aldrich, Saint Louis, Missouri, USA). Luria–Bertani medium (10 g/L tryptone, 10 g/L NaCl, and 5 g/L yeast extract, pH 7.4) was used for cloning purposes. Minimal medium (MM) used for growth and P(3HB) accumulation assays was modified from [28] and contains in g/L: KH_2_PO_4_ (0.39); (NH_4_)_2_SO_4_ (2.91); MgSO_4_·7H_2_O (0.31); CaCl_2_·2H_2_O (0.010); (NH_4_)_5_Fe(C_6_H_4_O_7_)_2_ (0.06); NaCl (1); trace elements solution (2 mL/L), which was prepared with H_3_BO_3_ (0.30 g/L); CoCl_2_·6H_2_O (0.20 g/L); ZnSO_4_·7H_2_O (0.10 g/L); MnCl_2_·4H_2_O (0.03 g/L); NaMoO_4_·2H_2_O (0.03 g/L); NiCl_2_·6H_2_O (0.02 g/L); CuSO_4_·5H_2_O (0.01 g/L). Use of xylose or glucose as carbon sources for MM is indicated as MMX and MMG, respectively. Sterile filtered solutions of arabinose or isopropyl *β*-d-1-thiogalactopyranoside (IPTG) were used as inducers at the indicated concentrations.

#### Bacterial strains and growth conditions

*Escherichia coli* DH10B (F− endA1 deoR+ recA1 galE15 galK16 nupG rpsL Δ(lac)X74 φ80lacZΔM15 araD139 Δ(ara,leu)7697 mcrA Δ(mrr-hsdRMS-mcrBC) StrR λ−) was used as a host for plasmid construction and propagation [29]. *Escherichia coli* cultures were grown in Luria–Bertani broth (200 rpm) or agar at 37 °C. When needed, the medium was supplemented with kanamycin (kan, 50 μg/mL). *Burkholderia sacchari* LMG 19450 LFM101 was used as the final host for pF8k-RFP and pF5k-RFP plasmids. Cells were grown at 30 °C, 150 rpm in MMG for pre inoculum to achieve higher biomass to begin the assay and MMX (5 g/L) for 24 h for growth assays and 15 g/L for 48 h for P(3HB) accumulation.

#### Bioinformatic analysis and primer design

The genes in the present study were selected using data from a recent analysis of *B. sacchari* genome from our group [30]. Minimum Tm of 60 °C and 18 bp hybridization to target was used as a standard for primer design. Primers were designed to amplify only the complete coding DNA sequence (CDS, from ATG to STOP codon) of the genes of interest and added the desired restriction site for cloning in the compatible BglBrick plasmid (Table 1). Synthetic ribosome binding sites (RBS) described elsewhere [19] were added in each forward primer to guarantee the efficient translation of the cloned sequences. When needed, a stop codon was added at the end of the corresponding CDS.

**Table 1:** Primers designed in the present study to amplify the genes of interest for cloning in the compatible BglBrick plasmid

Name	Sequence	Restriction site
*xylA*_F	AAAAAAGAATTC*TTTAAGAAGGAGATATACAT*ATGGGAGCCGAGCGCAAA	*Eco*RI
*xylB*_R	AAAAAAGGATCCTCAGGCGCCGGATGCGAATA	*Bam*HI
*xylF*_F	AAAAAACATATGAAGTTCGCCAAACGCCGTTCG	*Nde*I
*xylH*_R	AAAAAACTCGAGTCAGCGGCGGTTCGACCCC	*Xho*I
*xylE*_F	AAAAAAAGATCT*TTTAAGAAGGAGATATACAT*ATGACCGCGCTGGAAAAGAAAG	*Bgl*II
*xylE*_R	AAAAAACTCGAGTCAGTGTGAAAACCCGGAGGCCG	*Xho*I
*xylR*_F	AAAAAAAGATCT*TTTAAGAAGGAGATATACAT*ATGACCCGCGCCACCGCTTCCC	*BgI*II
*xylR*_R	AAAAAACTCGAGTCAGGAGTTTGATGTGCATGCGGCGCGC	*Xho*I
*Eco*RI-*Bgl*II_pF8K_F	AAAAAAGAATTCAAAAGATCT*TTTAAGAAGGAGATATACAT*ATGGCGAG	*Eco*RI, *Bgl*II
Prom_*Xba*I_pF8K_R	AAAAAATCTAGAGGAGAAACAGTAGAGAGTTGCGATAAAA	*Xba*I
*Xba*I_RBS_xylE_F	AAAAAATCTAGA*TTTAAGAAGGAGATATACAT*ATGACCGCGCTGGAAAAGAAAG	*Xba*I
*xylE*STOP_*Bgl*II_R	AAAAAAAGATCTTCAGTGTGAAAACCCGGAGGCCG	*Bgl*II
*Xba*I_RBS_*xylA*_F	AAAAAATCTAGA*TTTAAGAAGGAGATATACAT*ATGGGAGCCGAGCGCAAA	*Xba*I
*xylB*STOP_*Eco*RI_R	AAAAAAGAATTCTCAGGCGCCGGATGCGAATA	*Eco*RI
*Xba*I_RBS_*xylE*_F	AAAAAATCTAGA*TTTAAGAAGGAGATATACAT*ATGACCGCGCTGGAAAAGAAAG	*Xba*I
*xylE*STOP_*Bgl*II_R	AAAAAAAGATCTTCAGTGTGAAAACCCGGAGGCCG	*Bgl*II
*Xba*I_RBS_*xylA*_F	AAAAAATCTAGA*TTTAAGAAGGAGATATACAT*ATGGGAGCCGAGCGCAAA	*Xba*I
*xylB*STOP_*Eco*RI_R	AAAAAAGAATTCTCAGGCGCCGGATGCGAATA	*Eco*RI

Restriction sites added in the sequence are underlined

#### Gene amplification

Genes were amplified from the *B. sacchari* genome, using Q5^®^ High-Fidelity DNA Polymerase (New England Biolabs, Inc, Ipswich, Massachusetts, USA) in a Mastercycler Nexus Gradient model thermal cycler (Eppendorf AG, Hamburg, Germany) following the protocol recommended by the manufacturer. Cycling conditions were as follow: 98 °C 2′, 35X [98 °C 20″, 60 °C 20″, 72 °C (25″ per Kb)], 72 °C 5′.

#### Plasmid construction

Plasmids used in this study are listed in Table 2. pF backbones were constructed by amplifying pBBR1 ori from pBBR1MCS-2 plasmid [31] using pBBR1fullori_F and pBBR1fullori_R primers (Table 1). Purified PCR product was digested and ligated into pB8k-RFP and pB5k-RFP plasmids [19] using *Bcu*I and *Avr*II restriction sites. For construction of pF plasmids with xylose regulator (*xylR*), catabolism (*xylAB*), and transport genes (*xylFGH* and *xylE*), PCR products amplified with the indicated primers (Table 1) were isolated from agarose gel using GeneJET Gel Extraction and DNA Cleanup Micro Kit (Thermo Fisher Scientific Inc., Waltham, MA, USA) according to manufacturer’s instructions and cloned into pF8k-RFP and pF5k-RFP using the corresponding restriction enzymes (Table 1).

**Table 2:** Plasmids used in the present study

Name	ORI	Promoter	Resistance	Gene	Plasmid source
pBbB8k-RFP	BBR1^a^	pBAD	Kan	RFP	[19]
pBbB5k-RFP	BBR1^a^	placUV5	Kan	RFP	[19]
pBBR1MCS-2	BBR1	plac	Kan	lacZ	[39]
pF8k-RFP	BBR1	pBAD	Kan	RFP	Present study
pF5k-RFP	BBR1	placUV5	Kan	RFP	Present study
pF1k-GFP	BBR1	pTrc	Kan	GFP	Present study
pF6k-RFP	BBR1	pLlacO1	Kan	RFP	Present study
pF8k-*xylE*	BBR1	pBAD	Kan	*xylE*	Present study
pF8k-*xylFGH*	BBR1	pBAD	Kan	*xylFGH*	Present study
pF8k-*xylAB*	BBR1	pBAD	Kan	*xylAB*	Present study
pF8k-*xylR*	BBR1	pBAD	Kan	*xylR*	Present study
pF8k-*xylE*_RFP	BBR1	pBAD	Kan	*xylE,* RFP	Present study
pF8k-*xylFGH*_RFP	BBR1	pBAD	Kan	*xylFGH,* RFP	Present study
pF8k-*xylAB*_RFP	BBR1	pBAD	Kan	*xylAB,* RFP	Present study
pF5k-*xylR*	BBR1	placUV5	Kan	*xylR*	Present study

^a^Modified BBR1 as described [19]

#### *Burkholderia sacchari* transformation

Electrocompetent cells of *B. sacchari* were prepared according to previously established protocols. Briefly, 1 mL of culture in mid-exponential phase was inoculated into 50 mL of Luria–Bertani (250 mL flask) and incubated at 30 °C at 170 rpm until cells reached OD_600_ 0.6. The flask was incubated 15 min on ice, collected by centrifugation (15 min, 4500 rpm), washed twice with 50 mL of sterile water. The cell pellet was finally resuspended and aliquoted (50 µL each) in sterile 20% glycerol. Plasmids were transformed by electroporation using Gene Pulser Xcell™ electroporation system (Bio-Rad Laboratories, Inc. Hercules, California, USA). 100 ng of DNA were added to 50 µL electrocompetent cells and transferred to a 0.2 cm electroporation cuvette. Electroporation parameters were: 25 µF, 200 Ω, 2500 V.

#### Fluorescence and growth assays

Strains were inoculated in 120 µL of MMG and transferred to 96-well plate as pre inoculum. The plate was incubated 24 h at 30 °C and 170 rpm and diluted 1:100 in a 96-well plate with MMX. Absorbance at 600 nm was measured in the Synergy™ H1 96-well Hybrid Multi-Mode Reader (BioTek^®^ Instruments, Inc, Winooski, Vermont, USA). 96-well plates were incubated at 30 °C with continuous orbital agitation (548 cpm) in the Synergy H1 96-well plate reader. Growth (OD_600_) and fluorescence (RFP, exc: 535, emi: 620, gain 80) were monitored for 24 h (15 min intervals). Both parameters (fluorescence and growth) were measured in triplicate and data were expressed in arbitrary units, adjusted with the cell growth in each case.

#### P(3HB) accumulation assay

MM (1 g/L ammonium sulphate; xylose 15 g/L) was used to promote P(3HB) accumulation. 125 mL Erlenmeyer flasks containing 50 mL of MM were used. Initial optical density (OD_600_) was adjusted to 0.1 for all cultures. For the production analysis, two samples (10 mL each) were taken one at the beginning of the experiment and one after 48 h of culture (30 °C, 200 rpm). These samples were centrifuged (10,600×g, 4 °C, 10 min). Sugars were determined in the supernatant by liquid phase chromatography (HPLC) as described in “Analytical methods” section. The precipitated biomass was frozen and lyophilized to measure dry cell weight (CDW in g per liter) and to determine the composition of PHA by gas phase chromatography of propyl esters [32], as described in “Analytical methods” section.

### Analytical methods

#### Biomass concentration

10 mL of culture were harvested by centrifugation at 10,600×*g* and lyophilized in microtubes. In summary, samples were frozen at − 20 °C, transferred to a *FreeZone Triad Cascade Benchtop Freeze Dry System* (Labconco, Kansas City, Missouri, USA) and held at − 40 °C. After 2.5 h, the samples were maintained overnight at 0 °C and 0.080 mBar. After this period, the temperature was raised to 30 °C and the samples were taken from the apparatus. Dry biomass was weighed using an Adventurer Analytical Balance (Ohaus, Parsippany, New Jersey, USA) and expressed in grams per liter as cell dry weight (CDW).

#### Xylose determination

Xylose concentration was determined by liquid chromatography (HPLC) as previously described [24]. Briefly, 10 μL of each sample appropriately diluted were injected into a Ultimate 3000 HPLC (Dionex, Thermo Fisher Scientific Inc., Waltham, MA, USA) equipped with a Aminex HPX-87H Column (Bio-Rad Laboratories, Inc. Hercules, California, USA). For detection, a Refractive Index Detector Shodex RI-101 (Shodex, Munich, Germany) was used. Separation occurred at 45 °C with a H_2_SO_4_ solution (5 mM) at a flow rate of 0.6 mL/min. The standard curve was constructed using d(+)xylose solutions (Merck KGaA, Darmstadt, Germany) containing up to 3 g/L.

#### P(3HB) content and composition measurements

12 mg of freeze-dried cells were subjected to propanolysis [32] P(3HB) content was determined as described previously by Gomez [33] with an Agilent 7890A GC System (Agilent Technologies, Santa Clara, California, USA) equipped with an HP-5 capillary column (Agilent Technologies, Santa Clara, California, USA) after sample split (1:25). Helium (0.8 mL/min) was used as carrier gas. Benzoic acid and P(3HB) (both from Sigma-Aldrich, Saint Louis, Missouri, USA) were used as internal and external standards, respectively.

## Results and discussion

### Adapting BglBrick vectors to use in *B. sacchari*

*Escherichia coli* is a widely used prokaryotic host for recombinant protein production and metabolic engineering [34]. However, since the 1990s *B. sacchari, Pseudomonas putida* and other Gram-negative bacteria have demonstrated enormous potential for bioproduction at an industrial scale. Despite its remarkable potential, the lack of appropriate molecular tools still limits the application of metabolic engineering or synthetic biology approaches in *B. sacchari*. Previously constructed broad-host-range plasmid vectors derived from IncP and IncW incompatibility groups have been shown to be able to replicate in *Burkholderia* species. However, most research on *Burkholderia* species has focused on studying multi-resistant clinical isolates [27, 35, 36], and therefore, appropriate reporter systems, inducible promoters and other synthetic biology essential tools are not available for *B. sacchari.*

**Fig. 1:**
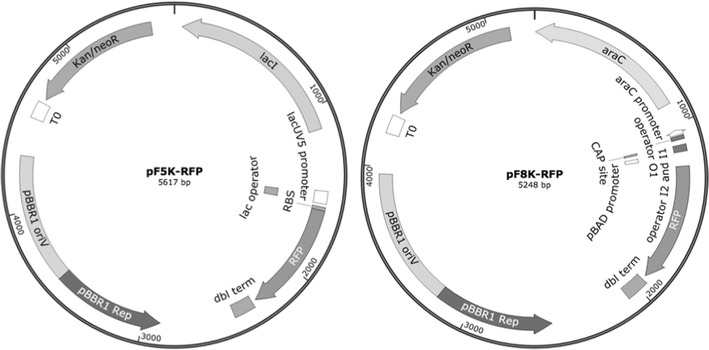
pF8k-RFP and pF5k-RFP plasmids, two Bglbrick-based expression vectors for *B. sacchari.* pF8k-RFP (5284 bp) and pF5k-RFP (5617 bp) vectors were constructed by changing the replication origins of BglBrick pB8k-RFP and pB5k-RFP plasmids, respectively. Nomenclature of these plasmids is identical to Bglbrick vectors, except the B (BBR1 from BglBrick vectors) is replaced by F (pBBR1 full from pBBR1MCS-2) (details in text). The figure depicts the origin of replication from pBBR1MCS-2, kanamycin resistance, red fluorescent protein RFP, pBAD expression system including BAD promoter and *E. coli araC*, placUV5 expression system including lacUV5 promoter and *E. coli lacI*

Aiming to engineer *B. sacchari* to improve both growth rate and P(3HB) production, two BglBrick vectors [19] (a commonly used expression platform available), were selected as a starting point. Since plasmids with the broad host range replication origin –pBBR1–, have been successfully transformed into *B. sacchari* [37], BglBrick plasmids containing pBBR1 (pB plasmids in BglBrick nomenclature) were first tested. Surprisingly, compared to the control plasmid pBBR1MCS-2, which produced around 2 × 10^3^ colonies/µg of plasmid DNA, transformation with pB8k-RFP plasmid resulted in no colonies. Aiming to understand this phenomenon and to generate a functional plasmid for *B. sacchari*, the sequences of both pBBR1 replication origins were compared. The pBBR1 ori, isolated from *Bordetella bronchiseptica* [38], encodes an essential replication protein (Rep) that shares sequence homology with other replication proteins present in Gram-negative bacteria [39]. In addition, the pBBR1 plasmid contains a series of sequences (direct repeats, putative IHF binding, and AT-rich regions) important for plasmid replication [40]. The sequence alignment of replication origins from pBBR1MCS-2 and BglBrick pBBR1 plasmids revealed significant differences that may explain the observed phenomenon. Specifically, a region of 198 bp containing sequences important for interaction with DnaA and other proteins relevant for plasmid replication and stable maintenance in Gram-negative bacteria [40, 41] is missing in the pBBR1 ori of the BglBrick plasmid. Furthermore, previous work has shown that differences in position, spacing, and arrangement of DnaA-binding sequences can significantly affect plasmid replication even in closely related Gram-negative bacteria [40]. In order to have a functional plasmid in *B. sacchari*, we constructed two vectors where the BBR1 replication origin of the BglBrick plasmids was replaced with the pBBR1 origin from the pBBR1MCS-2 plasmid, as described in “Materials and methods” section. The new plasmids preserve the same nomenclature as BglBrick vectors but replace the letter **B (**of BglBrick BBR1) with an **F**, representing the **f**ull pBBR1 origin from pBBR1MCS-2 (Fig. 1). Following this nomenclature, pF8k-RFP includes the replication of origin from pBBR1MCS-2 indicated by the letter F, a pBAD promoter indicated by the number 8 and the letter k representing kanamycin resistance. Transformation of *B. sacchari* with pF8k-RFP, pF5k-RFP, or pBBR1MCS-2 (as a control) produced a comparable number of colonies, indicating that the previously observed low transformation efficiency can be ascribed to the inefficient replication of the modified BglBrick pBBR1 in *B. sacchari*. In addition, our pF vectors also exhibited segregation stability comparable to that reported for pBBR1-MCS [27] in other *Burkholderia* species (data not shown).

### Inducible RFP expression in *B. sacchari*

**Fig. 2:**
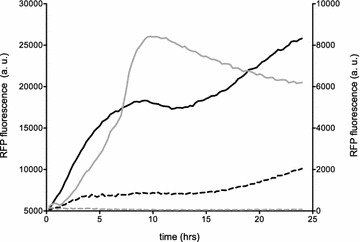
Time-dependent expression of RFP using plasmids pF8k-RFP and pF5k-RFP in *B. sacchari.* Late-exponential cells were diluted 100 times in MMX (with and without inducer) and transferred to 96-well plates. RFP fluorescence and optical density (OD_600_) were measured in triplicate every 15 min for 24 h. IPTG 0.5 mM (solid black line) or arabinose 5 mM (solid gray line) were used as inducers. Fluorescence was normalized by optical density and is expressed in arbitrary units. Uninduced cells are shown as dashed lines. Fluorescence profile of pF5k-RFP (black lines) is plotted on the left Y axis and pF8k-RFP (gray lines) on the right Y axis. All assays were performed in triplicate

Protein expression level is a fundamental parameter that often can be tuned for metabolic engineering purposes by modifying induction parameters (time, inducer concentration, etc.). Although pBAD and placUV5 promoters were used previously in non-enteric bacteria [42, 43] and other *Burkholderia* species [27], there is no information describing their use in *B. sacchari*. To address this gap in knowledge, we explored the effectiveness of both promoters, along with BglBrick RBS and terminator sequences for the expression of RFP in *B. sacchari,* using the plasmids pF8k-RFP and pF5k-RFP. As shown in Fig. 2, both promoters were able to drive expression of RFP in *B. sacchari,* additionally demonstrating that the synthetic RBS and terminator sequences of BglBrick vectors are also functional in *B. sacchari.*

**Fig. 3:**
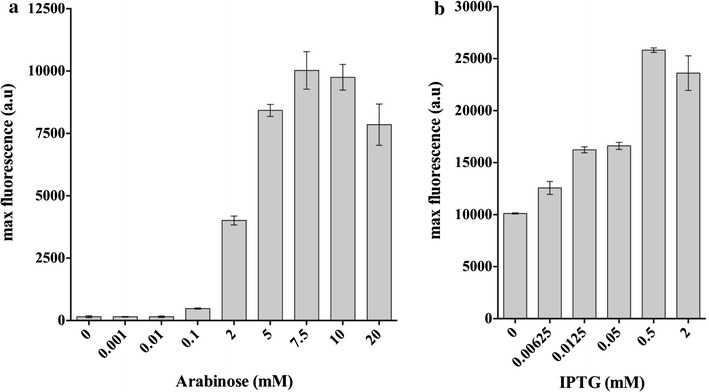
Inducer-dependent expression of pF8k-RFP and pF5k-RFP in *B. sacchari.* pF8k-RFP (**a**) and pF5k-RFP (**b**) cells in exponential growth phase, were diluted 100 times in MMX with increasing concentrations of inducer and transferred to 96-well plates. RFP fluorescence and optical density (OD 600 nm) were measured in triplicate every 15 min for 24 h. For each inducer concentration, maximum normalized fluorescence ± SD is shown

As shown in Figs. 2 and 3a, RFP expression from pF8k-RFP was strongly dependent on arabinose. Maximum pBAD induction reached 65-fold higher expression with 7.5 mM arabinose, compared to the uninduced control. Cultures induced with less than 0.1 mM arabinose were statistically indistinguishable (p = 0.05) from the uninduced control (Fig. 3a). While these data demonstrate inducible RFP expression from a pBAD promoter, we must also consider the possibility that because *B. sacchari* can efficiently catabolize arabinose this may also be utilized as a carbon source [21, 44]. For cultivation purposes, carbon sources are typically provided in a range of 0.2–0.5%, which is within the range of arabinose provided as inducer in *B. sacchari* (0.03–0.3%). Because arabinose can be catabolized, leading to changes in its concentration, induction kinetics can also fluctuate over time.

RFP expression was also effectively activated by pLacUV5 (Fig. 2), but in contrast to the strong inducer-dependence of pF8k-RFP, high background levels of fluorescence were observed in the strain transformed with pF5k-RFP under low induction levels (0.00625–0.0125 mM) and even in uninduced conditions (Fig. 3b). Maximum induction (2.5-fold) was reached with the addition of 0.5 mM IPTG. This result shows that while lacUV5 is functional, similar to *E. coli*, expression is leaky in *B. sacchari,* possibly due to its intrinsically higher ability to recruit RNA polymerase [19].

### *xylAB* overexpression improved growth rate in *B. sacchari*

**Fig. 4:**
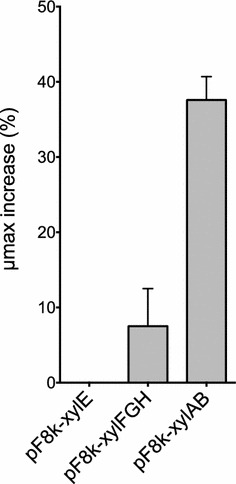
Expression of *xylAB* and *xylFGH* improve xylose utilization in *B. sacchari.* Late-exponential cells grown in MMG were diluted 1:100 in MMX with arabinose (7.5 mM) and transferred to 96-well plates. Optical density (OD_600_) was measured in triplicate every 15 min for 24 h. The μmax increase (%) was determined by comparing the growth rate of each strain with the one expressing RFP (pF8k-RFP)

Once the tools for controlling expression were characterized, we next sought to identify the best strategy to improve xylose utilization in *B. sacchari*. All characterized genes involved in xylose transport and catabolism were cloned and overexpressed using the pF8k plasmid. Strains were fully induced using 7.5 mM arabinose as described before (Fig. 3a). *Burkholderia sacchari* xylose utilization genes belonging to the xylose isomerase pathway including xylose transporters *xylE* (low-affinity xylose symporter) and *xylFGH* (ABC-type transporters ATP-dependent), and catabolic genes *xylA* (xylose isomerase) and *xylB* (xylulokinase), were cloned into pF8k using the restriction sites listed in Table 1. The pF8k plasmid was selected to clone the xylose genes to minimize basal expression and evaluate the role of proteins only when overexpressed. Confirmed plasmids carrying transporter or catabolic genes were transformed into *B. sacchari* and specific growth rate (µ_max_, h^−1^) for each strain was determined using GrowthRates software [45] from growth curves obtained in 96-well Synergy H1 plate reader (Biotek, Winooski, VT, USA). For growth assays, cells were pre-cultured in MMG and then transferred to MMX for the actual assay. As shown in Fig. 4, induction of pF8k-*xylAB* (7.5 mM arabinose) resulted in a 37.58% (0.205 h^−1^) growth rate increase compared to the control strain expressing RFP (0.149 h^−1^) with the same inducer concentration. Overexpression of *xylFGH* improved growth rate only marginally and the strain expressing *xylE* grew at a rate comparable to the control. The growth rate of all strains was comparable when uninduced (data not shown). With these results, it is tempting to suggest that unlike what has been observed in other microorganisms [18, 46, 47], in *B. sacchari,* the isolated overexpression of xylose transporters is not a strategy to substantially improve growth rate in xylose.

### Increased expression of *xylR* further improved growth rate and P(3HB) production

**Fig. 5:**
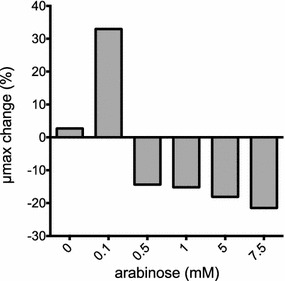
Low to medium expression of XylR improved growth rate of *B. sacchari.* Late-exponential cells grown in MMG were diluted 1:100 in MMX with increasing concentrations of arabinose (0–7.5 mM) and transferred to 96-well plates. Optical density (OD_600_ nm) was measured in triplicate every 15 min for 24 h. The μmax change (%) was determined by comparing the growth rate of each strain with the control strain expressing RFP induced with the same concentration of arabinose

In addition to transporters and catabolic genes, the transcriptional regulator XylR is a component of the xylose utilization *locus* in *B. sacchari*. Previously, overexpression of *xylR* in *E. coli* improved ethanol production from arabinose and xylose by increasing levels of XylA, XylB, XylF and XylG [48]. In order to coordinately increase xylose utilization and potentially improve P(3HB) production in *B. sacchari*, *xylR* was amplified and cloned into the pF8k plasmid to assess the effect of its overexpression on growth and P(3HB) production. As shown in Fig. 5 and Table 3, the growth rate of the pF8k-*xylR* strain increased by 33% (0.20 h^−1^) when induced with 0.1 mM arabinose, compared to a strain expressing RFP with the same inducer concentration. Surprisingly, the addition of more than 0.5 mM arabinose drastically impaired cell growth in MMX (Fig. 5). This result suggests that although increased expression of *xylR* enhanced xylose utilization, when both sugars are present, there is a certain point at which high levels of *xyl*R may somehow reduce arabinose assimilation, limiting growth rate when this carbon source is present at significant concentrations. Consistent with this hypothesis, arabinose concentration exhibited a clear positive correlation with growth rate when the control strain was grown in MMX supplemented with increasing arabinose concentrations for induction purposes (data not shown). The reduced growth rate observed when *xylR* was overexpressed may be attributed to a phenomenon called reciprocal regulation described in *E. coli* [48–50]. In this case, it has been demonstrated that XylR antagonizes AraC binding to target (Ara) promoters, thereby inhibiting expression of arabinose utilization genes. Although this mechanism has not been previously described in *B. sacchari*, the similar phenotype and sequence (61.1% similar to its *E. coli* counterpart) suggest that the mechanism observed in *E. coli* may explain the negative correlation observed here when increasing arabinose concentrations were used to induce *xylR* overexpression in *B. sacchari.*

**Table 3:** Summary of the results of P(3HB) production and xylose growth rate in *B. sacchari* overexpressing *xylR* or *rfp*

Plasmid	CDW (g/L)	%P(3HB)	P(3HB) (g/L)	µmax (h^−1^)	Y_xyl/P(3HB)_ (g/g)
pF8k-*xylR*	7.15 ± 0.58	65 ± 1.98	4.65	0.20 ± 0.003	0.348 ± 0.007
pF8k-RFP	5.12 ± 0.72	51.95 ± 3.01	2.66	0.156 ± 0.009	0.237 ± 0.005
pF5k-*xylR*	8.02 ± 0.44	71.07 ± 2.46	5.7	0.252 ± 0.01	0.396 ± 0.009
pF5k-RFP	5.34 ± 0.66	54 ± 2.88	2.89	0.162 ± 0.016	0.225 ± 0.006

Shake flask experiments with mineral medium supplemented with xylose (15 g/L). Cell dry weight (CDW), polymer content of CDW [P(3HB)], P(3HB) yield from xylose (Y_xyl/P(3HB)_), and P(3HB) volumetric productivity [P(3HB)] are presented. CDW was determined gravimetrically, carbohydrates by HPLC [49], and percentage of P(3HB) by gas chromatography of propyl esters [33]. Presented data correspond to averages from triplicate experiments. Standard deviation about those averages are also presented

**Fig. 6:**
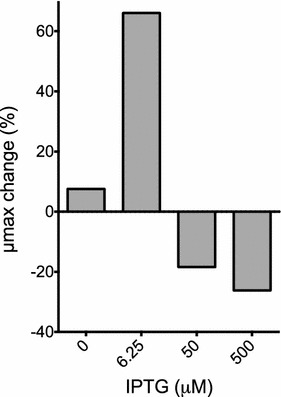
*xylR* expression affects the growth rate of *B. sacchari.* Late-exponential cells grown in MMG were diluted 1:100 in MMX with increasing concentrations of IPTG (0–500 µM) and transferred to 96-well plates. Optical density (OD_600_ nm) was measured in triplicate every 15 min for 24 h. The μmax change (%) was determined by comparing the growth rate of each strain with the control strain expressing RFP induced with the same concentration of IPTG

To avoid the use of arabinose and eliminate this variable (dual effect, as inducer and carbon source) from the experiment, we created the plasmid pF5k-*xylR*, where *xylR* expression is controlled by the addition of the non-metabolizable chemical analog of allolactose, Isopropyl β-d-1-thiogalactopyranoside (IPTG). Induction of *xylR* with low-level (6.25 µM IPTG) significantly increased growth rate (0.149–0.25 h^−1^, 66% increase) achieving the highest growth rate reported to date for *B. sacchari* using xylose as a carbon source. According to this result, low induction levels of *xylR* allowed the cells to adapt faster to the shift of carbon source (MMG was used as the carbon source in the pre-inoculum). Surprisingly, addition of higher IPTG concentrations (50 or 500 µM) delayed growth, even when xylose was used as a sole carbon source. Although the mechanism behind this phenomenon is unclear, these results correspond with observations in *E. coli,* which have demonstrated that high *xyl*R expression is deleterious to *E. coli* growth [48]. Regarding the direct effect of inducers on cell growth, it must be mentioned that although arabinose is used as a carbon source in *B. sacchari,* for each of the inducer concentrations shown in Figs. 4, 5 and 6, the cell growth improvement was calculated relative to the control strain (expressing RFP), induced with the same IPTG or arabinose concentration, and therefore, we have analyzed the effect of the indicated protein overexpression rather than the direct effect of the inducer. It is also important to mention that although arabinose 0.1 mM and IPTG 6.25 µM are in the low range, both concentrations caused significant increase of fluorescence (212.5 and 24.2% for arabinose and IPTG respectively), compared to the uninduced controls. Regarding the same issue for *xylR* expression induced by low inducer concentrations, due to the intrinsic differences of RFP and *xylR* coding sequences, it is not possible to directly use fluorescence levels to predict XylR concentrations in cells, however, based on the fluorescence data on Fig. 3, and the clear phenotypic differences (growth rate) observed in strains expressing *xylR*, it is likely to assume that for both promoters, even the addition of low concentration of inducer, significantly activates the expression of the corresponding protein causing the observed effects.

### Overexpression of *xylR* further improved P(3HB) accumulation

After demonstrating that *xylR* overexpression can improve growth on xylose, we next tested whether it can also impact P(3HB) production. P(3HB) accumulation was evaluated after 48 h in batch cultures, only for the best inducer concentration tested before (Fig. 6). As shown in Table 3, the strain expressing *xylR* under the control of a lacUV5 promoter induced with 6.25uM IPTG produced the highest P(3HB) titer, reaching 5.7 g/L (34% increase compared to the control strain expressing only RFP). This same strain generated the highest P(3HB) yield from xylose reported to date for *B. sacchari* (Y_P3HB/Xyl_ = 0.396 g/g), a 76% improvement compared to the control strain expressing RFP alone.

Prior to the results presented here, the highest reported P(3HB) yield from xylose was obtained from a wild-type *B. sacchari* strain which produced 0.29 g_P(3HB)_/g_xylose_ with a maximum growth rate of 0.21 h^−1^. However, it must be mentioned that this result was obtained when *B. sacchari* cells were grown with a glucose to xylose ratio of 1.0 [12]. Also emphasizing the importance of the present study, previous work from our group [2] determined the theoretical maximum conversion efficiency of xylose to P(3HB) using a *B. sacchari* metabolic model considering central metabolism, cellular biomass, and P(3HB) biosynthesis. The in silico analysis of elementary modes was performed in order to identify key metabolic modifications that would improve the xylose yield (Y_PHB/Xyl_) of *B. sacchari*. This analysis determined that the theoretical maximum xylose to P(3HB) yield is 0.40 g/g [2] In this work, the best strain (expressing *xylR* under the control of pLacUV5) achieved a yield of (0.396 g/g), reaching 92.3% of the theoretical yield.

## Conclusions

In this work we adapted two plasmids derived from BglBrick vectors allowing tunable protein expression in the non-model bacterium *Burkholderia sacchari*. In order to demonstrate its broad applicability and potential for bioproduction, we used these vectors to evaluate *B. sacchari* growth and P(3HB) accumulation in response to overexpression of all xylose isomerase pathway genes when using xylose as the sole carbon source. Overexpression of the xylose activated regulator *xylR,* significantly improved growth rate (0.162–0.252, 55% increase) and polymer yield (0.225–0.396, 76% increase) reaching 92.3% of the theoretical yield [2]. In addition, the marginal improvement observed when *xylFGH* or *xylE* transporters were overexpressed strongly suggests that contrary to what has been reported for *S. cerevisiae* [51–53], xylose transport is not the key factor limiting xylose utilization in *B. sacchari.* To the best of our knowledge, this is the first report demonstrating that *xylR* overexpression enhances growth rate and P(3HB) production in *B. sacchari*.

## Highlights

A. Two plasmids for tunable gene expression in *Burkholderia sacchari* are presented.
B. *xylR* overexpression significantly improved growth rate and polymer yield.
C. The highest P(3HB) yield reported from xylose in *B. sacchari* was obtained (71 %CDW)
